# Supplementary material for: Impact of Growth Conditions on High-Throughput Identification of Repurposing Drugs for Pseudomonas aeruginosa Cystic Fibrosis Lung Infections
Source: Antibiotics (Basel). 2024 Jul 12;13(7):642. doi: 10.3390/antibiotics13070642 (PMC11273527; doi:10.3390/antibiotics13070642)
Supplement: Supplementary file 1 [file antibiotics-13-00642-s001.zip › antibiotics-3038741-supplementary.pdf]

Article

Impact of Growth Conditions on High-Throughput Identification of Repurposing Drugs for *Pseudomonas aeruginosa* Cystic Fibrosis Lung Infections

Giovanni Di Bonaventura [1,2,*], Veronica Lupetti [1,2] and Arianna Pompilio [1,2]

[1] Department of Medical, Oral and Biotechnological Sciences, "G. d'Annunzio" University of Chieti-Pescara, 66100 Chieti, Italy; veronica.lupetti@studenti.unich.it (V.L.); arianna.pompilio@unich.it (A.P.)
[2] Center for Advanced Studies and Technology, "G. d'Annunzio" University of Chieti-Pescara, 66100 Chieti, Italy
* Correspondence: gdibonaventura@unich.it; Tel.: +39-0871-3554812

SUPPLEMENTAL MATERIAL

Table S1. Hits with potential activity towards *P. aeruginosa* RP73. Listed are 144 hits showing comparable activity under ENRICHED and CF-like experimental conditions.

				Growth reduction (% vs. CTRL) under:		
**Product Name**	**CAS No.**	**Research Area**	**Clinical Information**	**CF-like conditions**	**ENRICHED conditions**	**Hits with "antibacterial" indication**
Colistin (sulfate)	1264-72-8	Infection	Launched	100.0	100.0	X
Tyramine	51-67-2	Cardiovascular Disease	Phase 3	31.7	30.5	
6-Mercaptopurine	50-44-2	Cancer	Launched	23.2	25.4	
Camphor	76-22-2	Cancer; Infection; Inflammation/Immunology	Launched	24.8	23.4	
Chlorzoxazone	95-25-0	Inflammation/Immunology	Launched	30.3	30.7	
Pirfenidone	53179-13-8	Inflammation/Immunology; Cancer	Launched	33.4	33.5	
Amrinone	60719-84-8	Cardiovascular Disease	Launched	24.5	25.2	
Diethyltoluamide	134-62-3	Infection	Launched	27.1	30.6	
Methacholine (chloride)	62-51-1	Others	Launched	28.1	23.6	
Mecamylamine (hydrochloride)	826-39-1	Neurological Disease	Launched	21.9	24.6	
β-Elemene	515-13-9	Cancer	Phase 3	21.2	21.4	
Trapidil	15421-84-8	Cardiovascular Disease; Cancer	Launched	29.5	27.1	
Ciclopirox	29342-05-0	Infection; Cancer	Launched	100.0	99.3	
Sodium 4-aminosalicylate (dihydrate)	6018-19-5	Infection	Launched	24.2	24.9	X
Diazoxide	364-98-7	Cardiovascular Disease	Launched	23.0	21.3	
Nalidixic acid	389-08-2	Infection	Launched	100.0	98.2	X
Sulbactam	68373-14-8	Infection	Launched	100.0	99.9	X
Lomustine	13010-47-4	Cancer	Launched	26.1	23.2	
Sulfacetamide (Sodium)	127-56-0	Infection	Launched	45.2	46.7	X
β-Lapachone	4707-32-8	Cancer	Phase 2	28.1	27.6	
5-Azacytidine	320-67-2	Cancer; Infection	Launched	23.9	22.8	
Ribavirin	36791-04-5	Infection	Launched	33.7	36.2	
Epinastine	80012-43-7	Inflammation/Immunology; Endocrinology	Launched	20.8	21.6	
Levocarnitine propionate (hydrochloride)	119793-66-7	Others	Launched	27.5	28.7	
Daidzein	486-66-8	Endocrinology; Cancer	Launched	27.7	24.6	
AICAR	2627-69-2	Cancer	Phase 3	23.2	26.2	
Lofexidine	31036-80-3	Neurological Disease; Endocrinology	Launched	23.3	23.4	
T807	1415379-56-4	Neurological Disease	Phase 4	20.8	21.0	
Desvenlafaxine	93413-62-8	Neurological Disease	Launched	21.3	21.9	
Emixustat	1141777-14-1	Metabolic Disease	Phase 3	30.8	32.0	
Epetraborole (hydrochloride)	1234563-16-6	Infection	Phase 2	96.2	97.1	X
Chlormezanone	80-77-3	Neurological Disease	Launched	23.1	20.5	
Sulfamethazine	57-68-1	Infection	Launched	25.4	21.2	X
Niflumic acid	4394-00-7	Inflammation/Immunology	Launched	34.6	30.8	
Pramipexole (dihydrochloride)	104632-25-9	Neurological Disease	Launched	22.6	27.2	
Fisetin	528-48-3	Cancer; Metabolic Disease; Inflammation/Immunology; Neurological Disease	Phase 2	41.5	43.1	
Trimethoprim	738-70-5	Infection	Launched	41.0	37.1	X
Midodrine (hydrochloride)	43218-56-0	Neurological Disease	Launched	23.6	24.9	
Brimonidine	59803-98-4	Endocrinology	Launched	23.2	22.2	
Mebendazole	31431-39-7	Infection; Cancer	Launched	27.2	30.9	
Ibuprofen piconol	64622-45-3	Inflammation/Immunology	Launched	23.7	23.8	
Nortriptyline (hydrochloride)	894-71-3	Neurological Disease; Cancer	Launched	38.6	33.6	
Sodium gualenate	6223-35-4	Inflammation/Immunology	Launched	25.9	25.3	
Oxprenolol (hydrochloride)	6452-73-9	Cardiovascular Disease	Launched	24.9	26.9	
Gemcabene	183293-82-5	Metabolic Disease; Inflammation/Immunology	Phase 2	22.1	22.0	
Broxyquinoline	521-74-4	Infection	Launched	100.0	100.0	
Alcaftadine	147084-10-4	Inflammation/Immunology; Endocrinology	Launched	26.3	23.8	
BNC210	13589-06-5	Neurological Disease	Phase 2	31.5	35.1	
Zinc Pyrithione	13463-41-7	Cardiovascular Disease	Launched	100.0	100.0	X
Norfloxacin	70458-96-7	Infection	Launched	100.0	99.8	X
Mitotane	53-19-0	Cancer	Launched	38.8	35.5	
Gliclazide	21187-98-4	Cardiovascular Disease	Launched	30.0	30.2	
Cyproheptadine (hydrochloride)	969-33-5	Neurological Disease; Cardiovascular Disease	Launched	22.4	23.2	
Enoxacin (hydrate)	84294-96-2	Infection; Cancer	Launched	100.0	100.0	X
INCB054329	1628607-64-6	Cancer	Phase 2	25.3	29.9	
Cyproheptadine (hydrochloride sesquihydrate)	41354-29-4	Neurological Disease; Endocrinology	Launched	23.6	26.9	
SP-420	911714-45-9	Others	Phase 2	100.0	96.6	
Thiamphenicol	15318-45-3	Infection	Launched	100.0	100.0	X
Niraparib (hydrochloride)	1038915-64-8	Cancer	Launched	23.1	22.8	
Silver sulfadiazine	22199-08-2	Infection	Launched	100.0	98.5	X
Ethylenediaminetetraacetic acid (trisodium salt)	150-38-9	Others	Launched	34.4	38.0	
Florfenicol	73231-34-2	Infection	Launched	100.0	100.0	X
Capecitabine	154361-50-9	Cancer	Launched	24.5	25.5	
Ofloxacin	82419-36-1	Infection	Launched	95.2	100.0	X
Hydrocortisone	50-23-7	Inflammation/Immunology; Endocrinology; Cancer	Launched	20.5	23.4	
Efaproxiral (sodium)	170787-99-2	Cancer	Phase 3	27.2	25.7	
Ozenoxacin	245765-41-7	Infection	Launched	98.0	100.0	X
Cephalexin (monohydrate)	23325-78-2	Infection	Launched	28.0	29.6	X
Levofloxacin (hydrate)	138199-71-0	Infection	Launched	97.8	100.0	X
Gatifloxacin	112811-59-3	Infection	Launched	100.0	100.0	X
Droperidol	548-73-2	Neurological Disease	Launched	21.4	23.2	
Losmapimod	585543-15-3	Inflammation/Immunology; Cancer	Phase 3	30.0	34.4	
Resminostat (hydrochloride)	1187075-34-8	Cancer	Phase 2	48.8	46.4	
Lomefloxacin (hydrochloride)	98079-52-8	Infection	Launched	100.0	100.0	X
Valganciclovir (hydrochloride)	175865-59-5	Infection	Launched	25.2	29.4	
Sparfloxacin	110871-86-8	Infection	Launched	100.0	100.0	X
Naftopidil	57149-07-2	Cardiovascular Disease; Endocrinology	Launched	26.8	23.9	
Deoxycholic acid	83-44-3	Metabolic Disease	Launched	29.3	31.7	

Rubitecan	91421-42-0	Cancer	Phase 3	24.7	28.7	
Cinacalcet (hydrochloride)	364782-34-3	Cardiovascular Disease	Launched	38.0	41.1	
Quinine (hydrochloride dihydrate)	6119-47-7	Infection	Launched	24.3	23.2	
Diiodohydroxyquinoline	83-73-8	Infection	Launched	100.0	95.5	
Finafloxacin	209342-40-5	Infection	Launched	100.0	100.0	X
TAK-715	303162-79-0	Inflammation/Immunology	Phase 2	22.0	22.4	
Carvedilol	72956-09-3	Cancer; Inflammation/Immunology; Cardiovascular Disease	Launched	26.7	25.6	
Glafenine (hydrochloride)	65513-72-6	Inflammation/Immunology	Launched	23.0	22.2	
Gatifloxacin (hydrochloride)	121577-32-0	Infection	Launched	100.0	100.0	X
Pazufloxacin (mesylate)	163680-77-1	Infection	Launched	100.0	100.0	X
Trovafloxacin	147059-72-1	Infection	Launched	100.0	98.4	X
Aminophylline	317-34-0	Inflammation/Immunology	Launched	20.4	21.1	
Bendazac L-Lysine	81919-14-4	Others	Launched	21.9	24.6	
Cholic acid (sodium)	361-09-1	Metabolic Disease	Launched	20.8	22.6	
Samuraciclib (hydrochloride)	1805789-54-1	Cancer	Phase 2	36.6	34.1	
Pimasertib	1236699-92-5	Cancer	Phase 2	20.9	22.6	
PF-06459988	1428774-45-1	Cancer	Phase 2	24.3	20.7	
BX471	217645-70-0	Inflammation/Immunology; Endocrinology	Phase 2	33.4	33.7	
Aztreonam	78110-38-0	Infection	Launched	100.0	99.9	X
Sitafloxacin (hydrate)	163253-35-8	Infection	Launched	99.1	100.0	X
Vorolanib	1013920-15-4	Cancer; Cardiovascular Disease	Phase 3	20.7	23.6	
Ibrutinib (Racemate)	936563-87-0	Cancer	Launched	25.8	25.9	
Vilazodone carboxylic acid	163521-19-5	Others	Launched	24.6	26.6	
Ceftibuten (dihydrate)	118081-34-8	Infection	Launched	24.6	28.0	X
Gepotidacin	1075236-89-3	Infection	Phase 3	100.0	100.0	X
Diltiazem (hydrochloride)	33286-22-5	Cardiovascular Disease	Launched	29.7	31.7	
Betrixaban	330942-05-7	Cardiovascular Disease	Launched	34.1	32.1	
Danofloxacin (mesylate)	119478-55-6	Infection	Launched	100.0	100.0	X
AZD8186	1627494-13-6	Cancer	Phase 2	23.9	22.6	
Prulifloxacin	123447-62-1	Infection	Launched	100.0	100.0	X
Alogliptin (Benzoate)	850649-62-6	Metabolic Disease; Inflammation/Immunology; Cardiovascular Disease	Launched	30.5	28.7	
Tobramycin	32986-56-4	Infection	Launched	100.0	99.7	X
Givinostat (hydrochloride monohydrate)	732302-99-7	Cancer	Phase 3	60.0	57.7	
Methacycline (hydrochloride)	3963-95-9	Infection	Launched	100.0	100.0	X
Tetracycline (hydrochloride)	64-75-5	Infection	Launched	100.0	99.7	X
Tilorone (dihydrochloride)	27591-69-1	Infection; Cancer	Launched	25.4	25.2	
Avanafil	330784-47-9	Cardiovascular Disease	Launched	24.6	29.6	
Cefmetazole (sodium)	56796-39-5	Infection	Launched	33.0	33.5	X
Minocycline (hydrochloride)	13614-98-7	Infection	Launched	80.7	81.3	X
Tefinostat	914382-60-8	Cancer	Phase 2	67.7	69.3	
Selexipag	475086-01-2	Cardiovascular Disease; Endocrinology	Launched	42.1	47.1	
Oxytetracycline (hydrochloride)	2058-46-0	Infection	Launched	100.0	96.8	X
Latanoprostene bunod	860005-21-6	Neurological Disease	Launched	28.1	24.0	
Clebopride (malate)	57645-91-7	Metabolic Disease	Launched	27.2	25.6	
Chlortetracycline (hydrochloride)	64-72-2	Infection	Launched	100.0	100.0	X
Oprozomib	935888-69-0	Cancer	Phase 2	28.0	25.0	
Tenofovir alafenamide hemifumarate	1392275-56-7	Infection	Launched	20.4	22.5	
Puromycin (dihydrochloride)	58-58-2	Infection	Launched	30.9	29.5	X
SAR125844	1116743-46-4	Cancer	Phase 2	30.6	35.0	
Selumetinib (sulfate)	943332-08-9	Cancer	Launched	24.7	20.0	
Cefepime (Dihydrochloride Monohydrate)	123171-59-5	Infection	Launched	100.0	100.0	X
Vapendavir (diphosphate)	1198151-75-5	Infection; Inflammation/Immunology	Phase 2	24.2	27.9	
Cadazolid	1025097-10-2	Infection	Phase 3	100.0	100.0	X
Salmeterol (xinafoate)	94749-08-3	Inflammation/Immunology; Endocrinology	Launched	42.5	42.0	
Iotalamic acid	2276-90-6	Inflammation/Immunology	Launched	30.4	34.5	
Retaspimycin (Hydrochloride)	857402-63-2	Cancer	Phase 3	26.5	31.0	
Delafloxacin (meglumine)	352458-37-8	Infection	Launched	100.0	100.0	X
Evacetrapib	1186486-62-3	Cardiovascular Disease	Phase 3	28.1	26.5	
Icariin	489-32-7	Inflammation/Immunology; Cardiovascular Disease; Cancer	Phase 3	22.6	23.1	
Auranofin	34031-32-8	Cancer; Infection; Inflammation/Immunology	Launched	100.0	98.5	
Trametinib (DMSO solvate)	1187431-43-1	Cancer	Launched	20.0	21.4	
Meclocycline (Sulfosalicylate Salt)	73816-42-9	Infection	Launched	99.2	98.6	X
Rifamycin S	13553-79-2	Infection	Phase 3	99.1	100.0	X
Ceftaroline fosamil	400827-46-5	Infection	Launched	29.7	31.4	X
Cenicriviroc Mesylate	497223-28-6	Infection; Inflammation/Immunology; Endocrinology	Phase 3	28.1	32.3	
Rifapentine	61379-65-5	Infection	Launched	100.0	97.3	X

Table S2. Hits with potential activity towards *P. aeruginosa* RP73. Listed are 147 hits showing higher activity under CF-like than ENRICHED experimental conditions.

				Growth reduction (% vs. CTRL) under:		
**Product Name**	**CAS No.**	**Research Area**	**Clinical Information**	**CF-like conditions**	**ENRICHED conditions**	**Hits with "antibacterial" indication**
Dimercaprol	59-52-9	Infection	Launched	44.0	25.9	
N-Ethylmaleimide	128-53-0	Others	Phase 2	79.7	30.3	
5-Fluorouracil	51-21-8	Cancer	Launched	100.0	80.9	
Methylcobalamin	13422-55-4	Metabolic Disease	Launched	33.2	20.6	
Isoniazid	54-85-3	Infection	Launched	56.7	35.7	X
Dimetridazole	551-92-8	Infection	Launched	27.2	20.6	
Tavaborole	174671-46-6	Infection	Launched	100.0	53.8	
Tocofersolan	9002-96-4	Metabolic Disease	Launched	91.0	40.3	
Uric acid	69-93-2	Inflammation/Immunology	Phase 3	48.5	20.2	
Levetiracetam	102767-28-2	Neurological Disease	Launched	42.6	29.1	
Sulforaphane	4478-93-7	Cancer; Inflammation/Immunology	Phase 3	100.0	75.9	
Tirapazamine	27314-97-2	Cancer	Phase 3	100.0	65.0	
Meglumine	6284-40-8	Others	Launched	38.1	32.6	
3-AP	143621-35-6	Cancer	Phase 3	97.4	83.4	
Pargyline (hydrochloride)	306-07-0	Cancer; Cardiovascular Disease	Launched	30.8	24.4	
Selenomethionine	1464-42-2	Cancer	Launched	66.4	36.4	
PRIMA-1Met	5291-32-7	Cancer	Phase 3	43.6	38.4	
Medetomidine	86347-14-0	Neurological Disease; Endocrinology	Launched	53.4	24.2	
(R)-(-)-Ibuprofen	51146-57-7	Inflammation/Immunology; Cancer	Launched	43.9	21.5	
Toloxatone	29218-27-7	Neurological Disease	Launched	43.6	24.7	
Mirogabalin	1138245-13-2	Neurological Disease	Launched	47.3	24.3	
Sulfacetamide	144-80-9	Infection	Launched	90.9	29.8	X
Clofibric acid	882-09-7	Others	Launched	64.4	35.5	
Gluconate (Calcium)	299-28-5	Others	Launched	27.6	20.9	
Aniracetam	72432-10-1	Neurological Disease	Launched	42.6	24.4	
Prilocaine	721-50-6	Neurological Disease	Launched	30.5	24.4	
Acetazolamide	59-66-5	Cardiovascular Disease; Cancer	Launched	59.9	28.5	
Detomidine (hydrochloride)	90038-01-0	Neurological Disease; Endocrinology	Launched	28.6	21.9	
Furazolidone	67-45-8	Infection	Launched	100.0	93.4	X
Resveratrol	501-36-0	Cancer; Infection; Inflammation/Immunology	Launched	100.0	82.4	
Lamivudine	134678-17-4	Infection	Launched	29.5	22.2	
Solriamfetol (hydrochloride)	178429-65-7	Neurological Disease	Launched	55.8	31.2	
Tetrahydrobiopterin	17528-72-2	Inflammation/Immunology	Launched	37.1	24.5	
Uridine	58-96-8	Others	Launched	36.6	23.1	
Floxuridine	50-91-9	Cancer; Infection	Launched	42.2	31.6	
Isoprenaline (hydrochloride)	51-30-9	Cardiovascular Disease; Endocrinology	Launched	38.6	29.1	
Triflusal	322-79-2	Inflammation/Immunology	Launched	37.1	21.5	
Sulfamethoxazole	723-46-6	Infection	Launched	100.0	73.5	X
S-(+)-Ketoprofen	22161-81-5	Inflammation/Immunology	Launched	37.9	26.2	
Carmofur	61422-45-5	Cancer	Launched	100.0	36.7	
Thalidomide	50-35-1	Cancer; Inflammation/Immunology	Launched	30.7	24.9	
Salsalate	552-94-3	Inflammation/Immunology	Launched	34.8	23.2	
Tolfenamic Acid	13710-19-5	Inflammation/Immunology; Cancer	Launched	61.5	30.1	
Cinoxacin	28657-80-9	Infection	Launched	100.0	39.1	X
Melperone	3575-80-2	Neurological Disease	Phase 3	32.4	22.7	
Sulfamerazine	127-79-7	Infection	Launched	73.4	46.6	X
RRx-001	925206-65-1	Cancer; Infection; Inflammation/Immunology	Phase 3	100.0	24.8	
Sulfamethizole	144-82-1	Infection	Launched	66.6	59.2	X
Sulfadiazine (sodium)	547-32-0	Infection	Launched	86.2	49.4	X
Adefovir	106941-25-7	Infection	Launched	28.6	22.5	
Tolcapone	134308-13-7	Neurological Disease	Launched	100.0	45.8	
Ebselen	60940-34-3	Cancer; Infection; Inflammation/Immunology; Neurological Disease	Phase 3	100.0	59.1	
Phenytoin (sodium)	630-93-3	Neurological Disease	Launched	50.6	23.6	
Nifuroxazide	965-52-6	Cancer; Infection	Launched	48.4	25.0	X
Sulfathiazole (sodium)	144-74-1	Infection	Launched	68.7	40.7	X
Sulfisomidin	515-64-0	Infection	Launched	49.9	40.8	X
Sulfamonomethoxine	1220-83-3	Infection	Launched	100.0	45.8	X
Sulfalene	152-47-6	Infection	Launched	67.5	29.9	X
Flufenamic acid	530-78-9	Inflammation/Immunology	Launched	72.0	36.1	
Pridopidine	346688-38-8	Neurological Disease	Phase 3	32.4	22.1	
Sulfachloropyridazine	80-32-0	Infection	Launched	83.7	62.9	X
Pizotifen	15574-96-6	Neurological Disease	Launched	41.1	31.2	
Ticlopidine (hydrochloride)	53885-35-1	Cardiovascular Disease	Launched	43.7	34.8	
Flumazenil	78755-81-4	Neurological Disease	Launched	27.3	20.0	
Clioquinol	130-26-7	Infection; Cancer	Launched	92.9	72.1	
Nitazoxanide	55981-09-4	Infection; Cancer	Launched	57.8	50.1	
Nimustine (hydrochloride)	55661-38-6	Cancer	Launched	69.4	26.7	
Sulfadimethoxine	122-11-2	Infection	Launched	82.3	41.0	X
Avobenzone	70356-09-1	Others	Launched	41.5	28.0	
5-(N,N-Hexamethylene)-amiloride	1428-95-1	Cancer; Infection	Phase 3	41.8	25.0	
Sulfaphenazole	526-08-9	Infection	Launched	100.0	37.1	X
Centhaquin	57961-90-7	Inflammation/Immunology; Cardiovascular Disease	Phase 3	68.7	40.7	
Sulfadimethoxine (sodium)	1037-50-9	Infection	Launched	91.3	31.3	X
Duloxetine (hydrochloride)	136434-34-9	Neurological Disease	Launched	59.6	39.4	
Chloroquinoxaline sulfonamide	97919-22-7	Cancer; Infection	Phase 2	47.0	20.8	
CC-115	1228013-15-7	Cancer	Phase 2	29.4	23.4	
Pumosetrag Hydrochloride	194093-42-0	Metabolic Disease	Phase 3	49.6	27.0	
Omeprazole	73590-58-6	Cancer; Infection; Metabolic Disease	Launched	35.4	26.3	

Panobinostat	404950-80-7	Cancer	Launched	99.7	67.8	
Dextrorotation nimorazole phosphate ester	1124347-33-6	Infection	Launched	46.8	22.1	X
Pafuramidine	186953-56-0	Infection	Phase 3	84.9	31.6	
Tedizolid	856866-72-3	Infection	Launched	46.8	27.2	X
Transcrocetinate disodium	591230-99-8	Cancer	Phase 2	47.6	38.4	
Vicagrel	1314081-53-2	Cardiovascular Disease	Phase 2	55.3	35.9	
Bromfenac (sodium hydrate)	120638-55-3	Inflammation/Immunology	Launched	52.8	21.3	
Fosfluconazole	194798-83-9	Infection	Launched	50.8	30.1	
Erlotinib	183321-74-6	Cancer	Launched	36.6	21.3	
Deracoxib	169590-41-4	Inflammation/Immunology	Launched	54.4	40.8	
Fursultiamine	804-30-8	Inflammation/Immunology; Neurological Disease	Launched	36.8	22.8	
PAT-1251	2007885-39-2	Inflammation/Immunology	Phase 2	34.1	21.9	
Mericitabine	940908-79-2	Infection	Phase 2	37.9	29.6	
Prednisone acetate	125-10-0	Inflammation/Immunology; Endocrinology	Launched	52.8	30.6	
Pritelivir	348086-71-5	Infection	Phase 2	38.8	24.8	
Verubecestat	1286770-55-5	Neurological Disease	Phase 3	38.2	29.1	
JNJ-42165279	1346528-50-4	Neurological Disease	Phase 2	43.0	35.7	
Ceralasertib	1352226-88-0	Cancer	Phase 2	58.8	47.4	
Opicapone	923287-50-7	Neurological Disease	Launched	65.9	39.6	
Spironolactone	52-01-7	Metabolic Disease; Cancer	Launched	37.8	30.4	
Bavisant (dihydrochloride hydrate)	1103522-80-0	Neurological Disease; Endocrinology	Phase 2	52.3	31.9	
Vismodegib	879085-55-9	Cancer	Launched	32.7	25.4	
Camicinal	923565-21-3	Metabolic Disease	Phase 2	27.8	21.3	
ACT-709478	1838651-58-3	Neurological Disease	Phase 2	75.5	35.5	
Pantoprazole (sodium hydrate)	164579-32-2	Cancer; Inflammation/Immunology	Launched	43.4	37.1	
Mycophenolate Mofetil	128794-94-5	Cancer	Launched	50.6	24.0	
Rivaroxaban	366789-02-8	Cardiovascular Disease	Launched	65.8	31.6	
Elenbecestat	1388651-30-6	Neurological Disease	Phase 3	36.9	30.3	
Doripenem (monohydrate)	364622-82-2	Infection	Launched	100.0	46.7	X
Pravastatin (sodium)	81131-70-6	Cardiovascular Disease; Cancer	Launched	33.0	23.6	
Bictegravir	1611493-60-7	Infection	Launched	89.4	38.4	
Tedizolid (phosphate)	856867-55-5	Infection	Launched	52.0	34.1	X
Crizotinib	877399-52-5	Cancer	Launched	31.5	23.7	
BTRX-335140	2244614-14-8	Metabolic Disease	Phase 2	39.8	21.2	
Belotecan (hydrochloride)	213819-48-8	Cancer	Launched	46.8	24.6	
Imisopasem manganese	218791-21-0	Cancer	Phase 2	72.2	52.6	
Nedisertib	1637542-33-6	Cancer	Phase 2	37.9	24.1	
Verapamil (hydrochloride)	152-11-4	Cardiovascular Disease	Launched	32.5	22.1	
Epothilone B	152044-54-7	Cancer; Infection	Phase 3	45.8	27.2	
Fimepinostat	1339928-25-4	Cancer	Phase 2	69.9	64.7	
LY2090314	603288-22-8	Cancer	Phase 2	31.6	25.6	
Dexamethasone phosphate disodium	2392-39-4	Inflammation/Immunology; Endocrinology	Launched	53.4	29.5	
Fusidic acid (sodium salt)	751-94-0	Infection	Launched	60.5	38.6	X
Piperacillin (sodium)	59703-84-3	Infection	Launched	100.0	73.0	X
XL-784	1224964-36-6	Cardiovascular Disease	Phase 2	91.4	58.3	
Merestinib	1206799-15-6	Cancer	Phase 2	44.9	29.5	
Thiocolchicoside	602-41-5	Inflammation/Immunology; Neurological Disease	Phase 4	36.2	25.3	
Cinaciguat	329773-35-5	Cardiovascular Disease	Phase 2	49.8	35.8	
Telcagepant	781649-09-0	Neurological Disease	Phase 3	43.0	26.2	
KW-2478	819812-04-9	Cancer	Phase 2	50.5	25.0	
PCI-27483	871266-63-6	Cancer	Phase 2	84.4	78.2	
Cinaciguat (hydrochloride)	646995-35-9	Cardiovascular Disease	Phase 2	36.4	24.8	
Mirodenafil (dihydrochloride)	862189-96-6	Others	Launched	31.9	22.6	
Novobiocin (Sodium)	1476-53-5	Infection; Cancer	Launched	97.2	80.0	X
DTP3 (TFA)		Cancer	Phase 4	45.3	31.2	
Cefoperazone	62893-19-0	Infection	Launched	100.0	62.9	X
Alvespimycin (hydrochloride)	467214-21-7	Cancer	Phase 2	48.7	22.3	
Flumatinib (mesylate)	895519-91-2	Cancer	Phase 3	48.7	24.1	
Sulbutiamine	3286-46-2	Neurological Disease	Launched	69.6	20.8	
Pictilisib (dimethanesulfonate)	957054-33-0	Cancer	Phase 2	34.4	27.1	
Transcrocetin meglumine salt		Cancer	Phase 2	67.0	22.6	
Rifamycin (sodium)	14897-39-3	Infection	Launched	96.9	86.0	X
Edoxaban (tosylate)	480449-71-6	Cardiovascular Disease	Launched	40.6	33.1	
Troxerutin	7085-55-4	Cancer; Metabolic Disease; Inflammation/Immunology; Neurological Disease	Launched	31.7	20.2	
Merbromin	129-16-8	Others	Launched	100.0	70.0	X
Sultamicillin (tosylate)	83105-70-8	Infection	Launched	100.0	79.4	X
(±)-Bisoprolol (hemifumarate)	104344-23-2	Inflammation/Immunology; Endocrinology	Launched	46.8	31.2	
Rifampicin	13292-46-1	Infection	Launched	98.9	92.3	X
Piperaquine (tetraphosphate tetrahydrate)	915967-82-7	Infection	Launched	58.7	36.4	

Table S3. Hits with potential activity towards *P. aeruginosa* RP73. Listed are 229 hits showing higher activity under ENRICHED than CF-like experimental conditions.

				Growth reduction (% vs. CTRL) under:		
**Product Name**	**CAS No.**	**Research Area**	**Clinical Information**	**CF-like conditions**	**ENRICHED conditions**	**Hits with "antibacterial" indication**
Punicalagin	65995-63-3	Cancer; Infection; Metabolic Disease	Phase 4	71.4	94.0	
Polymyxin B (Sulfate)	1405-20-5	Infection	Launched	55.8	100.0	X
Methyl Salicylate	119-36-8	Inflammation/Immunology	Launched	23.4	32.0	
Vigabatrin (hydrochloride)	1391054-02-6	Neurological Disease	Launched	39.3	94.5	
6-Thioguanine	154-42-7	Cancer; Infection; Inflammation/Immunology	Launched	40.3	56.4	
Tannic acid	1401-55-4	Cancer; Inflammation/Immunology	Launched	72.7	100.0	
4-Methylumbelliferone	90-33-5	Cancer; Cardiovascular Disease	Launched	24.7	40.3	
Cotinine	486-56-6	Others	Phase 4	22.2	35.0	
Propofol	2078-54-8	Neurological Disease	Launched	22.5	64.1	
Aspirin	50-78-2	Inflammation/Immunology; Cancer	Launched	21.9	55.6	
Theophylline	58-55-9	Cancer	Launched	32.5	39.7	
Apronal	528-92-7	Neurological Disease	Launched	23.2	33.1	
Dalbavancin (hydrochloride)	2227366-51-8	Infection	Launched	21.1	62.4	X
Carbimazole	22232-54-8	Endocrinology	Launched	21.0	30.1	
Nitroxoline	4008-48-4	Infection; Cancer	Launched	92.4	100.0	X
Isosorbide mononitrate	16051-77-7	Cardiovascular Disease; Cancer	Launched	23.9	32.2	
Bronopol	52-51-7	Infection	Launched	92.0	100.0	X
Tegafur	17902-23-7	Cancer	Launched	31.2	49.8	
Etidronic acid	2809-21-4	Others	Launched	30.2	46.9	
Betahistine (dihydrochloride)	5579-84-0	Endocrinology; Inflammation/Immunology; Neurological Disease	Launched	28.5	38.2	
Felbinac	5728-52-9	Inflammation/Immunology	Launched	31.7	45.2	
Chloroxine	773-76-2	Infection	Launched	37.3	99.0	X
Sulfaguanidine	57-67-0	Infection	Launched	39.1	45.8	X
5-Hydroxytryptophan	56-69-9	Metabolic Disease	Launched	27.5	42.0	
Chlorquinaldol	72-80-0	Infection	Launched	53.0	100.0	X
Levamisole (hydrochloride)	16595-80-5	Infection; Neurological Disease	Launched	26.4	52.6	
Flurbiprofen	5104-49-4	Inflammation/Immunology; Cancer	Launched	29.2	41.0	
Diflunisal	22494-42-4	Inflammation/Immunology; Cancer	Launched	40.2	51.7	
Setiptiline	57262-94-9	Neurological Disease	Launched	28.8	60.1	
Pralidoxime (iodide)	94-63-3	Neurological Disease	Launched	31.5	42.5	
Ciclopirox (olamine)	41621-49-2	Infection; Cancer	Launched	26.9	100.0	
Pardoprunox (hydrochloride)	269718-83-4	Neurological Disease; Endocrinology	Phase 3	31.9	39.9	
Baicalein	491-67-8	Cancer	Launched	33.8	47.3	
Alpha-Estradiol	57-91-0	Inflammation/Immunology	Launched	22.3	41.6	
Flutamide	13311-84-7	Cancer	Launched	20.4	40.2	
Oleic acid	112-80-1	Cancer; Metabolic Disease	Launched	22.5	58.2	
Guanabenz (Acetate)	23256-50-0	Cardiovascular Disease; Endocrinology	Launched	20.2	62.8	
Selgantolimod	2004677-13-6	Infection	Phase 2	27.6	43.8	
Embelin	550-24-3	Cancer	Launched	32.2	86.8	
Diclofenac	15307-86-5	Inflammation/Immunology	Launched	29.0	34.6	
Disulfiram	97-77-8	Metabolic Disease; Cancer	Launched	31.5	77.9	
Piroctone olamine	68890-66-4	Infection	Launched	91.8	97.7	
Etifoxine	21715-46-8	Neurological Disease	Phase 3	35.5	77.7	
Ellagic acid	476-66-4	Cancer; Neurological Disease	Phase 2	23.1	61.8	
Safinamide	133865-89-1	Neurological Disease	Launched	24.3	39.7	
Neticonazole	130726-68-0	Cancer; Infection	Launched	24.4	51.5	
Eicosapentaenoic Acid	10417-94-4	Neurological Disease; Cancer	Launched	28.1	53.3	
Arachidonic acid	506-32-1	Inflammation/Immunology	Phase 4	23.6	30.6	
(-)-Epigallocatechin	970-74-1	Cancer	Phase 4	22.6	40.7	
Tafamidis	594839-88-0	Neurological Disease	Launched	25.0	41.0	
Nimesulide	51803-78-2	Inflammation/Immunology	Launched	23.3	37.1	
Tipepidine (hydrochloride)	1449686-84-3	Neurological Disease	Phase 2	28.2	46.1	
Benorilate	5003-48-5	Inflammation/Immunology; Endocrinology	Launched	27.5	40.5	
Pregnenolone	145-13-1	Neurological Disease	Launched	21.5	79.9	
Belinostat	866323-14-0	Cancer	Launched	42.7	58.3	
Asenapine (hydrochloride)	1412458-61-7	Neurological Disease	Launched	25.2	37.3	
Remetinostat	946150-57-8	Cancer	Phase 2	27.9	42.6	
Senicapoc	289656-45-7	Others	Phase 3	21.5	69.4	
Cyclopentolate (hydrochloride)	5870-29-1	Neurological Disease	Launched	20.3	29.1	
Docosahexaenoic Acid	6217-54-5	Neurological Disease	Launched	25.4	35.6	
ASP-9521	1126084-37-4	Cancer	Phase 2	30.4	54.2	
SQ109	502487-67-4	Infection	Phase 2	21.8	53.5	X
Ciprofloxacin	85721-33-1	Infection	Launched	65.4	100.0	X
Triprolidine (hydrochloride monohydrate)	6138-79-0	Inflammation/Immunology; Neurological Disease	Launched	30.0	41.5	
E7820	289483-69-8	Cancer	Phase 2	25.1	31.4	
N-(p-amylcinnamoyl) Anthranilic Acid	110683-10-8	Cardiovascular Disease	Phase 2	24.5	59.2	
Idebenone	58186-27-9	Neurological Disease	Launched	23.8	63.9	
Neticonazole (hydrochloride)	130773-02-3	Cancer; Infection	Launched	47.2	90.9	
Gemcabene (calcium)	209789-08-2	Metabolic Disease; Inflammation/Immunology	Phase 2	24.3	32.9	
Dapoxetine (hydrochloride)	129938-20-1	Neurological Disease	Launched	23.3	32.9	
Ethacridine (lactate)	1837-57-6	Infection	Launched	24.9	83.3	X
Clotrimazole	23593-75-1	Infection; Cancer	Launched	23.8	76.0	
Fluoxetine (hydrochloride)	56296-78-7	Neurological Disease; Cancer	Launched	25.8	56.5	
Silmitasertib	1009820-21-6	Cancer	Phase 2	37.2	43.2	
Raxatrigine hydrochloride	934240-31-0	Neurological Disease	Phase 2	30.0	48.5	
Amorolfine (hydrochloride)	78613-38-4	Infection	Launched	31.4	39.3	
Bithionol	97-18-7	Cancer	Launched	86.8	100.0	
Estradiol valerianate	979-32-8	Endocrinology; Cancer	Launched	27.8	63.1	

Fezolinetant	1629229-37-3	Endocrinology	Phase 3	25.8	33.9	
Nitrendipine	39562-70-4	Cardiovascular Disease	Launched	21.3	28.5	
Nadifloxacin	124858-35-1	Infection	Launched	93.2	100.0	X
Ixazomib	1072833-77-2	Cancer	Launched	82.4	87.7	
Levofloxacin	100986-85-4	Infection	Launched	37.9	100.0	X
Ganetespib	888216-25-9	Cancer	Phase 3	30.5	48.8	
Quinestrol	152-43-2	Endocrinology; Cancer	Launched	24.3	50.3	
Curcumin	458-37-7	Cancer	Phase 4	54.9	100.0	
BMS-690514	859853-30-8	Cancer; Cardiovascular Disease	Phase 2	22.9	29.7	
Cinnarizine	298-57-7	Cardiovascular Disease; Endocrinology	Launched	23.8	55.4	
Incyclinide	15866-90-7	Cancer	Phase 2	44.6	91.9	
Silmitasertib (sodium salt)	1309357-15-0	Cancer	Phase 2	28.1	33.5	
Lornoxicam	70374-39-9	Inflammation/Immunology	Launched	22.9	41.5	
Deferasirox	201530-41-8	Cancer	Launched	52.9	68.6	
Entinostat	209783-80-2	Cancer	Phase 3	20.9	27.2	
Vortioxetine (hydrobromide)	960203-27-4	Neurological Disease	Launched	23.2	83.3	
Tebipenem	161715-21-5	Infection	Phase 3	92.9	100.0	X
Bortezomib	179324-69-7	Cancer	Launched	26.3	46.1	
Elafibranor	923978-27-2	Metabolic Disease	Phase 3	35.2	42.8	
Ilginatinib	1239358-86-1	Cancer	Phase 2	21.3	34.4	
Abiraterone acetate	154229-18-2	Cancer	Launched	21.3	54.8	
Indacaterol	312753-06-3	Neurological Disease; Endocrinology	Launched	20.0	67.4	
Perhexiline maleate	6724-53-4	Cancer; Cardiovascular Disease	Launched	31.7	59.7	
Clascoterone	19608-29-8	Endocrinology	Phase 3	34.3	41.0	
Cabotegravir	1051375-10-0	Infection	Phase 4	74.9	86.1	
BPN14770	1606974-33-7	Neurological Disease	Phase 2	52.1	58.8	
Tosedostat	238750-77-1	Cancer	Phase 2	24.2	40.0	
Miltefosine	58066-85-6	Infection; Cancer	Launched	23.8	40.8	
Decloxizine (dihydrochloride)	13073-96-6	Inflammation/Immunology; Endocrinology	Launched	32.3	46.1	
Amiselimod (hydrochloride)	942398-84-7	Cancer	Phase 2	60.6	81.0	
Bretylium (tosylate)	61-75-6	Neurological Disease	Launched	30.7	36.6	
Mefloquine (hydrochloride)	51773-92-3	Infection	Launched	40.4	75.2	
Donepezil (Hydrochloride)	120011-70-3	Neurological Disease	Launched	20.1	25.3	
Tegaserod (maleate)	189188-57-6	Metabolic Disease; Neurological Disease	Launched	23.3	100.0	
AT7519 (Hydrochloride)	902135-91-5	Cancer	Phase 2	20.4	39.5	
Dolutegravir	1051375-16-6	Infection	Launched	41.3	56.4	
Bromperidol	10457-90-6	Neurological Disease	Launched	21.4	49.7	
Obeticholic acid	459789-99-2	Others	Launched	26.2	51.1	
Bepridil (hydrochloride hydrate)	74764-40-2	Cardiovascular Disease	Launched	42.0	65.3	
Fludrocortisone acetate	514-36-3	Inflammation/Immunology	Launched	33.8	52.7	
AB928	2239273-34-6	Inflammation/Immunology	Phase 2	22.0	27.4	
Pimavanserin	706779-91-1	Neurological Disease	Launched	33.6	53.9	
SB-705498	501951-42-4	Neurological Disease	Phase 2	37.1	42.6	
Uprosertib	1047634-65-0	Cancer	Phase 2	33.9	51.5	
Pefloxacin (mesylate)	70458-95-6	Infection	Launched	37.3	100.0	X
PRT062607 (Hydrochloride)	1370261-97-4	Cancer	Phase 2	45.3	58.1	
Miransertib	1313881-70-7	Cancer; Infection	Phase 2	22.9	47.3	
Kasugamycin (hydrochloride hydrate)	200132-83-8	Infection	Launched	20.3	71.8	X
Rabusertib	911222-45-2	Cancer	Phase 2	28.7	82.8	
Fipronil	120068-37-3	Neurological Disease	Launched	23.2	31.7	
Alobresib	1637771-14-2	Cancer	Phase 2	35.3	44.5	
Alpelisib	1217486-61-7	Cancer	Launched	21.6	27.6	
Eltrombopag	496775-61-2	Cardiovascular Disease; Cancer	Launched	62.2	99.5	
Canagliflozin	842133-18-0	Metabolic Disease; Cancer	Launched	24.1	62.9	
Chloramphenicol succinate (sodium)	982-57-0	Metabolic Disease; Infection	Launched	43.2	78.7	X
Lomibuvir	1026785-55-6	Infection	Phase 2	30.3	53.3	
Loxapine (succinate)	27833-64-3	Neurological Disease	Launched	40.6	54.5	
Buparlisib (Hydrochloride)	1312445-63-8	Cancer	Phase 3	28.2	43.4	
Gefitinib	184475-35-2	Cancer	Launched	39.6	50.6	
(S)-Crizotinib	1374356-45-2	Cancer	Launched	23.9	77.4	
GW 501516	317318-70-0	Metabolic Disease; Cancer	Phase 4	21.1	53.2	
Cenerimod	1262414-04-9	Inflammation/Immunology	Phase 2	33.9	67.7	
PF-04457845	1020315-31-4	Neurological Disease	Phase 2	49.5	77.8	
Ipatasertib	1001264-89-6	Cancer	Phase 3	21.3	31.2	
(-)-Epigallocatechin Gallate	989-51-5	Cancer	Phase 4	41.8	70.1	
Terazosin (hydrochloride dihydrate)	70024-40-7	Endocrinology; Metabolic Disease	Launched	30.7	37.0	
Oxytetracycline	79-57-2	Infection	Launched	90.6	98.7	X
Edicotinib	1142363-52-7	Inflammation/Immunology; Neurological Disease	Phase 2	24.4	48.0	
Ebastine	90729-43-4	Inflammation/Immunology; Endocrinology	Launched	32.8	77.5	
Terfenadine	50679-08-8	Cancer; Inflammation/Immunology; Endocrinology	Launched	38.5	70.3	
Flunarizine (dihydrochloride)	30484-77-6	Neurological Disease	Launched	22.6	61.0	
ARQ 531	2095393-15-8	Cancer; Inflammation/Immunology	Phase 2	25.4	64.0	
MK-0773	606101-58-0	Endocrinology	Phase 2	26.0	32.8	
Gemifloxacin (mesylate)	210353-53-0	Infection	Launched	55.2	100.0	X
Zoliflodacin	1620458-09-4	Infection	Phase 3	92.8	100.0	X
Rolapitant	552292-08-7	Cancer; Endocrinology	Launched	43.6	53.6	
Demeclocycline (hydrochloride)	64-73-3	Infection	Launched	41.7	99.5	X
Tafamidis meglumine	951395-08-7	Neurological Disease	Launched	23.1	45.9	
BMS-986020 (sodium)	1380650-53-2	Metabolic Disease; Inflammation/Immunology	Phase 2	25.8	42.6	
Ruboxistaurin (hydrochloride)	169939-93-9	Metabolic Disease	Phase 3	27.0	63.7	

Chlorhexidine	55-56-1	Infection	Launched	30.6	100.0	X
Indacaterol (maleate)	753498-25-8	Endocrinology	Launched	24.2	54.4	
Ripretinib	1442472-39-0	Cancer	Launched	24.2	30.2	
α-Tocopherol (phosphate)	38976-17-9	Others	Phase 4	39.2	53.3	
Doxycycline (hyclate)	24390-14-5	Infection	Launched	31.9	51.7	X
Temocapril (hydrochloride)	110221-44-8	Cardiovascular Disease	Launched	20.2	26.5	
Gossypol	303-45-7	Cancer	Phase 3	39.7	100.0	
BI 2536	755038-02-9	Cancer	Phase 2	37.4	43.5	
Azatadine (dimaleate)	3978-86-7	Inflammation/Immunology; Endocrinology	Launched	20.5	34.9	
Penfluridol	26864-56-2	Neurological Disease; Cancer	Launched	20.5	42.6	
Gliquidone	33342-05-1	Metabolic Disease	Launched	20.4	33.0	
Vecabrutinib	1510829-06-7	Cancer	Phase 2	20.4	35.1	
JNJ-18038683	851376-05-1	Neurological Disease	Phase 2	31.2	39.5	
(+)-Ketoconazole	142128-59-4	Infection	Launched	20.4	75.2	
Azeliragon	603148-36-3	Neurological Disease	Phase 3	20.2	81.5	
Garenoxacin (Mesylate hydrate)	223652-90-2	Infection	Launched	85.1	100.0	X
Cefozopran (hydrochloride)	113981-44-5	Infection	Launched	42.0	98.8	X
Globalagliatin	1234703-40-2	Metabolic Disease	Phase 2	24.2	53.5	
Estramustine (phosphate sodium)	52205-73-9	Cancer; Endocrinology	Launched	32.0	52.3	
Eltrombopag (Olamine)	496775-62-3	Cardiovascular Disease; Cancer	Launched	43.0	97.9	
Lumateperone (tosylate)	1187020-80-9	Neurological Disease	Launched	21.0	61.9	
Amlodipine (besylate)	111470-99-6	Cardiovascular Disease	Launched	45.3	54.7	
Lefamulin (acetate)	1350636-82-6	Infection	Launched	27.8	58.3	X
Motesanib (Diphosphate)	857876-30-3	Cancer	Phase 3	21.9	34.0	
Bosentan (hydrate)	157212-55-0	Cardiovascular Disease; Endocrinology	Launched	23.6	33.2	
S-Adenosyl-L-methionine (tosylate)		Cancer; Inflammation/Immunology; Neurological Disease	Launched	26.7	32.8	
Gossypol (acetic acid)	12542-36-8	Cancer	Phase 3	60.6	81.7	
(R)-(-)-Gossypol acetic acid	866541-93-7	Cancer	Phase 2	62.9	85.5	
Deserpidine	131-01-1	Metabolic Disease; Neurological Disease	Launched	34.7	55.8	
Tafenoquine (Succinate)	106635-81-8	Infection	Launched	31.0	51.8	
Tigecycline	220620-09-7	Infection; Cancer	Launched	43.3	52.7	X
Omadacycline (hydrochloride)	1196800-39-1	Infection	Launched	51.4	83.1	X
Tosufloxacin (tosylate hydrate)	1400591-39-0	Infection	Launched	81.2	100.0	X
Toremifene (citrate)	89778-27-8	Cancer; Infection	Launched	34.1	56.2	
Montelukast (sodium)	151767-02-1	Inflammation/Immunology	Launched	33.2	42.9	
Candesartan Cilexetil	145040-37-5	Cardiovascular Disease; Endocrinology	Launched	40.1	73.1	
Visomitin	934826-68-3	Inflammation/Immunology	Launched	30.5	100.0	
Cefoselis (sulfate)	122841-12-7	Infection	Launched	33.9	83.3	X
Octenidine (dihydrochloride)	70775-75-6	Infection	Launched	31.1	100.0	X
Eravacycline (dihydrochloride)	1334714-66-7	Infection	Launched	91.8	98.1	X
Cloperastine fendizoate	85187-37-7	Inflammation/Immunology	Launched	24.7	46.0	
Tazemetostat (hydrobromide)	1467052-75-0	Cancer	Launched	24.2	37.6	
Teniposide	29767-20-2	Cancer	Launched	25.8	31.7	
Fucoxanthin	3351-86-8	Cancer; Metabolic Disease; Inflammation/Immunology	Phase 2	20.0	41.7	
Valemetostat (tosylate)	1809336-93-3	Cancer	Launched	29.0	36.4	
AZD-5991	2143061-81-6	Cancer	Phase 2	22.6	42.9	
Manidipine (dihydrochloride)	89226-75-5	Cardiovascular Disease	Launched	21.1	33.1	
Sisomicin (sulfate)	53179-09-2	Infection	Launched	93.9	100.0	X
Aramchol	246529-22-6	Metabolic Disease	Phase 3	21.6	40.9	
Dihydroergocristine (mesylate)	24730-10-7	Neurological Disease	Launched	24.6	62.8	
Afatinib (dimaleate)	850140-73-7	Cancer	Launched	28.9	46.8	
Ritonavir	155213-67-5	Infection	Launched	24.7	63.4	
Dabigatran etexilate (mesylate)	872728-81-9	Cardiovascular Disease	Launched	35.1	55.0	
TMC647055 (Choline salt)		Infection	Phase 2	38.9	47.1	
Erythromycin	114-07-8	Infection	Launched	33.7	88.9	X
Asunaprevir	630420-16-5	Infection	Launched	35.6	55.8	
Azithromycin	83905-01-5	Infection; Cancer	Launched	39.1	89.7	X
Telotristat etiprate	1137608-69-5	Neurological Disease; Cancer	Launched	22.7	51.9	
Davercin	55224-05-0	Infection	Launched	22.0	77.2	X
Pentagastrin	5534-95-2	Cancer; Endocrinology	Launched	21.7	51.9	
Cobicistat	1004316-88-4	Infection	Launched	20.4	30.8	
Gamithromycin	145435-72-9	Infection	Launched	25.2	91.2	X
Rifaximin	80621-81-4	Infection	Launched	61.1	100.0	X
GSK2838232	1443460-91-0	Infection	Phase 2	26.5	45.1	
Telithromycin	191114-48-4	Infection	Launched	29.3	44.1	X
Josamycin	16846-24-5	Infection	Launched	39.6	82.0	X
Rifabutin	72559-06-9	Infection	Launched	20.1	100.0	X
Cangrelor (tetrasodium)	163706-36-3	Inflammation/Immunology; Cardiovascular Disease	Launched	20.2	50.5	
Saroglitazar Magnesium	1639792-20-3	Metabolic Disease	Phase 3	22.7	49.2	
Deslanoside	17598-65-1	Cardiovascular Disease	Launched	21.2	36.8	
Tigecycline (tetramesylate)		Infection; Cancer	Launched	62.2	97.9	X
